# Microstructural Evolution and Mechanical Properties of SiC/Al-40Si Composites Fabricated by High Pressure Solidification

**DOI:** 10.3390/ma16124312

**Published:** 2023-06-11

**Authors:** Rong Zhang, Chunming Zou, Zunjie Wei, Hongwei Wang

**Affiliations:** 1 School of Materials Science and Engineering, Fujian University of Technology, Fuzhou 350118, China; zhang_r1992@163.com; 2 School of Materials Science and Engineering, Harbin Institute of Technology, Harbin 150001, China; wanghw@hit.edu.cn

**Keywords:** solidification, high pressure, mechanical properties, sic/al-40si composites, primary Si

## Abstract

The microstructure and mechanical properties of SiC/Al-40Si composites prepared under high pressure were studied. As the pressure increases from 1 atm to 3 GPa, the primary Si phase in the Al-40Si alloy is refined. With increasing pressure, the composition of the eutectic point increases, the solute diffusion coefficient decreases exponentially, and the concentration of Si solute at the front of the solid–liquid interface of the primary Si is low, which contributes to the refining of the primary Si and inhibiting its faceted growth. The bending strength of SiC/Al-40Si composite prepared under 3 GPa was 334 MPa, which was 66% higher compared to the Al-40Si alloy prepared under the same pressure.

## 1. Introduction

Aluminum–silicon (Al-Si) alloys are characterized by low density, high specific strength, low thermal expansion and high thermal conductivity, and have great potential for widespread use in aerospace, automotive and electronics applications. Al-Si alloys exhibit different properties based on their Si content. Al-Si alloys with low Si content (less than 13%) exhibit excellent formability and are commonly prepared through the die casting process, and are widely utilized in the aerospace and automotive industries for the production of components and casings [1]. Al-Si alloys with medium Si content (13–20%) demonstrate favorable heat resistance and wear resistance, which make them suitable for high-temperature and high-pressure (HP) conditions, and are frequently employed in the manufacturing of engine pistons [2]. Al-Si alloys with a high Si content (more than 20%) possess a low coefficient of thermal expansion and excellent thermal conductivity, and are commonly employed in the field of electronic packaging, including components such as heat sinks and packaging bases [3].

When the Si content is more than 20%, coarse primary Si is easily formed, which leads to stress concentration and reduces the strength of the composite [4]. Establishing how to eliminate the adverse effect of the coarse primary Si is the key to improving the properties of high-Si Al alloys. Adding chemical elements, reinforcement and improving the preparation process are all effective ways to improve the properties of high-Si Al alloys. The strengthening of Al-Si alloys by means of the addition of chemical elements mainly includes the refinement of primary Si using modifiers such as P [5], P + Ce, B, and Sr [6], as well as the formation of AlSi_2_Sc_2_, Al_2_Si_2_La, and NbSi_2_ precipitated phases via the addition of Sc, La, and Nb elements [7]. SiC [8] and Al_2_O_3_ [9] are the most common reinforcements for strengthening Al-Si alloys. At present, the main preparation methods include spray deposition, powder metallurgy, semi-solid forming, pressure/pressureless infiltration and hot pressing [10,11,12,13]. However, the pressure of the above preparation method is generally less than 100 MPa, the bonding strength between the microstructures is not high, and there are some pore defects [14].

Pressure can change the distance between atoms, causing changes in crystal structures and generating new states of materials, so high-pressure technology has great potential for the preparation of new high-performance materials [15,16,17,18,19,20,21,22]. It is important to explore the thermodynamic and kinetic changes, microstructural evolution and strengthening mechanisms under HP conditions. Generally, an increase in solidification pressure can change the eutectic point [23], increase the solubility of elements [24], and improve interfacial stability [25]. For Al-Si alloys, we have developed mathematical models to analyze the effect of pressure on the morphological evolution of the Al phase (spherical → cellular → dendritic) [26] and the Al-Si eutectic spacing [27]. However, there are fewer theoretical studies and experimental results on the effect of pressure on primary Si. The microstructural evolution and refinement mechanisms of primary Si under HP are still key questions to be elucidated.

In metal matrix composites, reinforcements can be divided into two types: ex situ and in situ. We obtained in situ SiC/Al-38.6Si composites via the in situ formation of SiC in the Al-40Si-1C system under HP, and its bending strength was increased by 60.3% compared to that of Al-40Si alloy [28]. However, the holding time required for in situ synthesis of about 3 vol% SiC under HP is 30 min, which is long and costly, and it is difficult to produce a high volume fraction of SiC via an in situ reaction. Therefore, this paper attempts to prepare SiC/Al-40Si composites by means of ex situ 20 vol% SiC and shortening the holding time to 5 min.

In this work, the Al-40Si alloys were prepared under HP, the microstructural evolution of the primary Si was investigated, and a mathematical model was developed to analyze the effect of pressure on the size and morphology of the primary Si. In addition, the 20 vol.% SiC/Al-40Si composite was prepared using ex situ SiC particles under HP, and the SiC-Si spherical microstructure formed by the primary Si and the network-distributed SiC was investigated, while the mechanical properties and strengthening mechanism of the composite were studied.

## 2. Experimental Procedures

**Figure 1:**
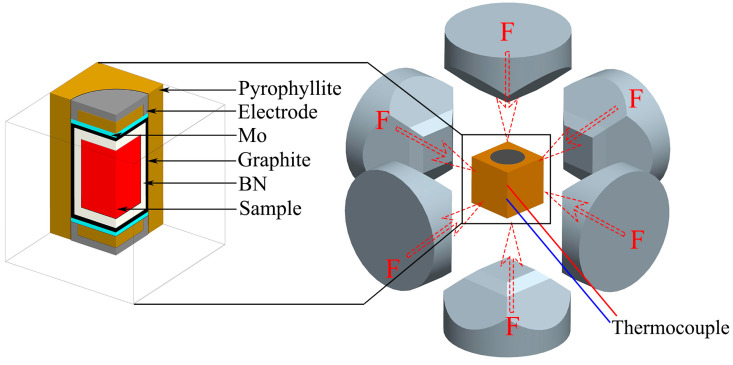
HP solidification experiment. Adapted with permission from Ref. [28]. Copyright 2021, Elsevier.

Al-40 wt%Si alloy powder (TiJO Metal Co., Ltd., Changsha, China, nitrogen gas atomization, 9–11 μm) and SiC particles (Xingrongyuan Technology Co., Ltd., Beijing, China, 0.1–1 μm) were used as raw materials. The powders were mixed at a volume ratio of 8:2 of Al-40Si alloy powders to SiC particles and mixed together in a ball mill at 90 rpm for 6 h. Al-40Si alloy powder and SiC/Al-40Si mixed powder were compressed into cylinders with a diameter of 20 mm and a height of 18 mm at a pressure of 300 MPa. The samples were then placed in a boron nitride (BN) crucible and then placed in a graphite crucible. The graphite crucible was sealed in pyrophyllite with electrodes and molybdenum (Mo) sheets at both ends. The HP conditions were achieved by squeezing with a six-anvil, as shown in Figure 1. The heating temperature was measured using a B-type platinum-rhodium thermocouple (Chuanyi Co., Ltd., Chongqing, China). The equipment pressure was calibrated with the room temperature phase transition point of Bi (I-II transition at 2.55 GPa). The pressure was first increased to the required pressure (1–3 GPa), and then the sample was heated to the required temperature (1273–1373 K) and held for 5 min. After that, the heating was stopped and cooled to room temperature at a cooling rate of 20 K/s, and finally the pressure was released.

The samples were ground with SiC paper and polished with a diamond solution. A 0.5 vol% HF solution was used to etch the samples for 10–15 s. The microstructure was characterized using an OLYMPUS optical microscope (OM, Japan) and ZEISS scanning electron microscope (SEM, Germany) equipped with energy dispersive X-ray spectroscopy (EDS, Germany). Three-point bending tests were conducted on the resulting samples using an Instron 3382 tester (USA) with a sample size of 2 mm × 3 mm × 19 mm and a bending speed of 0.3 mm/min.

## 3. Results

### 3.1. Microstructure

**Figure 2:**
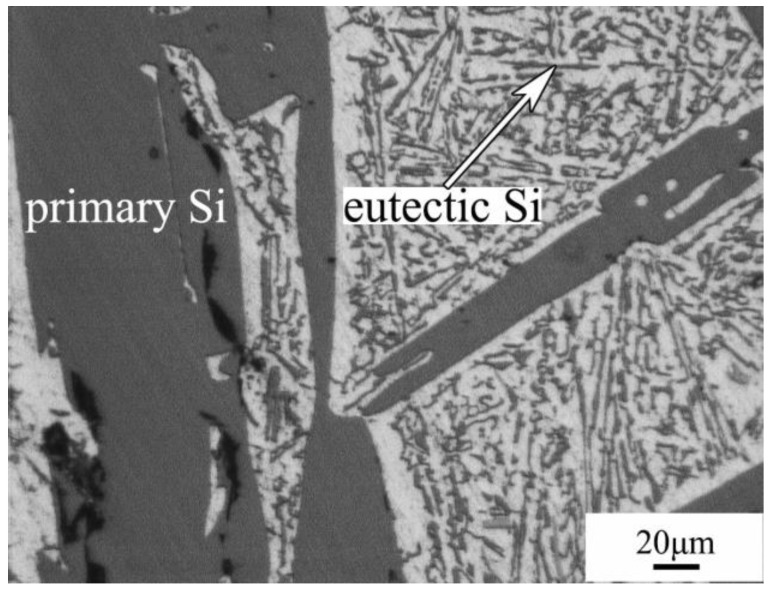
Microstructures of the Al-40Si alloy fabricated under 1 atm.

The microstructure of the Al-40Si alloy fabricated under 1 atm is shown in Figure 2. The morphology of the primary Si fabricated under atmospheric pressure is coarse lath-shaped, showing the characteristics of small plane growth.

**Figure 3:**
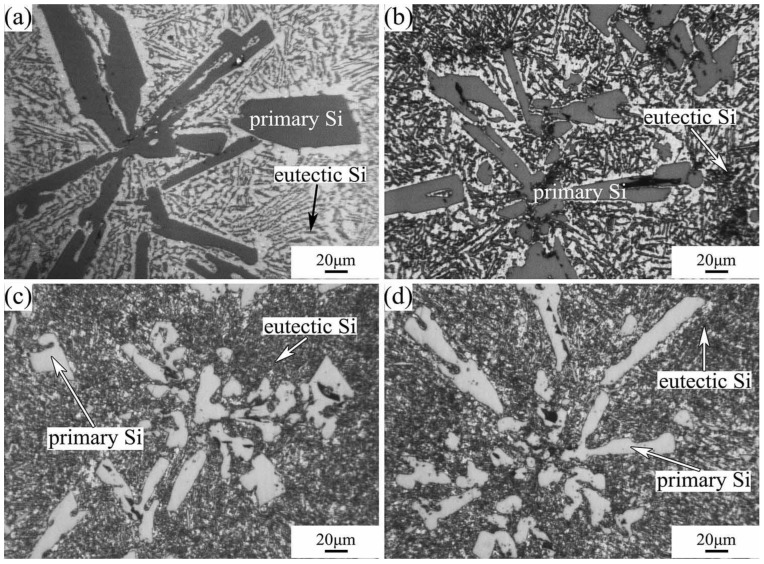
Microstructures of Al-40Si alloys fabricated under HP: (**a**) 1373 K/1 GPa; (**b**) 1323 K/2 GPa; (**c**) 1273 K/3 GPa; (**d**) 1373 K/3 GPa.

The microstructures of Al-40Si alloys fabricated under HP are shown in Figure 3. With the increase in pressure, the morphology of the primary Si phase gradually changes from coarse lath-like and five-petal star-like to fine strip-like and block-like. Meanwhile, the volume fraction of primary Si decreases, while the volume fraction of Al-Si eutectic phase increases. Additionally, the Al-Si eutectic spacing decreases with increasing pressure, which is consistent with our previous results obtained for the Al-20Si alloy [27]. As the solidification pressure increases, the primary Si is refined, and the sharp angle of the primary Si becomes blunt, and the growth morphology of the primary Si changes from faceted to non-faceted. At a pressure of 3 GPa, the primary Si and Al-Si eutectic did not change significantly as the melting temperature increased from 1273 K to 1373 K.

### 3.2. Microstructure Evolution Mechanism

**Figure 4:**
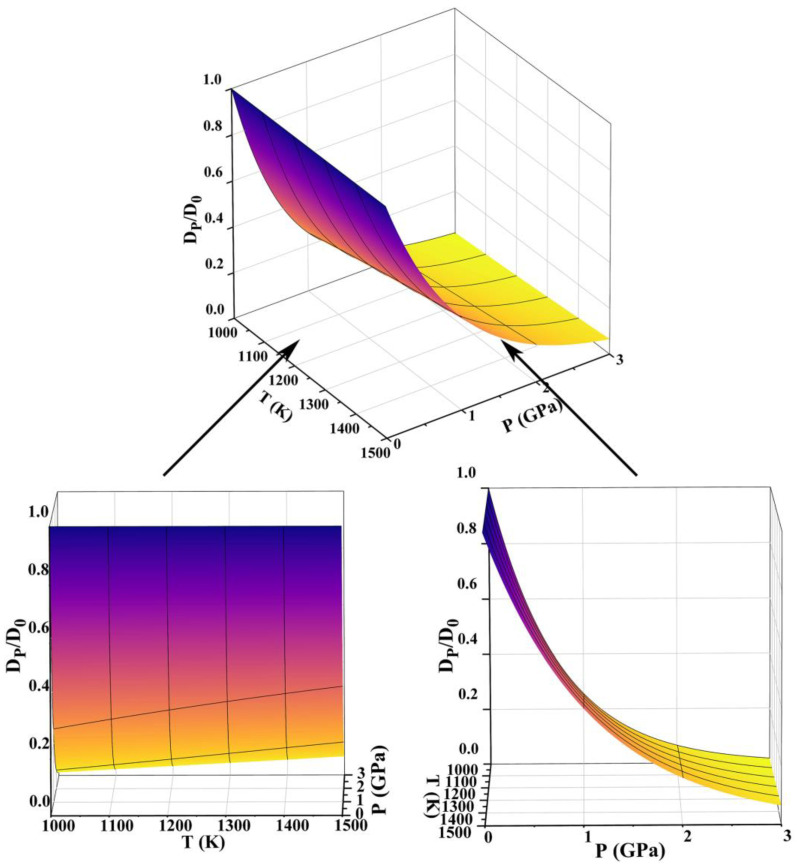
Relationship between pressure, temperature and solute diffusion coefficient of Al-40Si alloy.

The solute diffusion coefficient has a significant impact on the growth process of the primary Si. The ratio of the solute diffusion coefficient at HP and atmospheric pressure can be expressed by the following Equation [29]:

(1) $$\frac{D_{P}}{D_{0}}=\exp\left[-\frac{V_{m}^{l}\Delta P}{RT}\right]$$

where $V_{m}^{l}$ is the liquid molar volume, *D_P_* is the solute diffusion coefficient at HP, *D*_0_ is the solute diffusion coefficient at atmospheric pressure, Δ*P* is the change in pressure from HP to atmospheric pressure, and *R* is the gas constant. For Al-40Si alloy, $V_{m}^{l}$ = 1.13 × 10^−5^ m^3^/mol [30]. As shown in Figure 4, the calculation was performed according to Equation (1) to obtain the *D_P_*/*D*_0_ in the Al-40Si alloy as a function of pressure and temperature.

From the figure, it can be found that an increase in pressure causes an exponential decrease in the solute diffusion coefficient. Additionally, the increase in temperature from 1000 K to 1500 K causes a linear increase in the solute diffusion coefficient, but the increase is smaller. The influence of pressure on the solute diffusion coefficient is dominant in the calculated pressure and temperature range.

**Figure 5:**
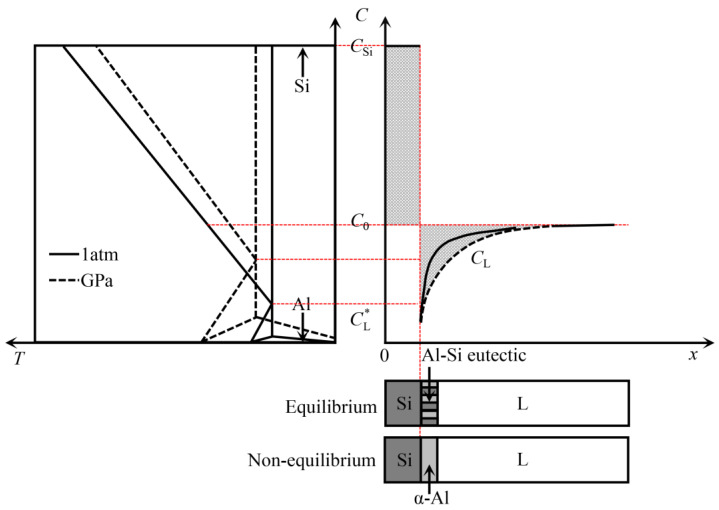
Schematic illustration of solute redistribution during the growth of primary Si in Al-Si alloy prepared under different pressures.

To further investigate the refinement mechanism of high-pressure solidification on the primary Si, the solute distribution at the growth interface front of the primary Si was investigated. Figure 5 shows a schematic illustration of the solute redistribution during the growth of primary Si at different pressures. Assuming that the solidus and the liquidus in the Al-Si phase diagram are straight lines, the solid–liquid (S–L) interface is in thermodynamic equilibrium, there is no diffusion in the solid phase and only diffusion in the liquid phase, and the solute distribution at the S–L interface front can be expressed as [31]:

(2) $$C_{L}=C_{0}+(C_{L}^{*}-C_{0})\exp(-\frac{V}{D}x)$$

where *C*_L_ is the liquid phase composition of the Al-Si alloy, *C*_0_ is the initial composition of the Al-Si alloy, $C_{L}^{*}$ is the liquid phase solute composition at the front of the S–L interface, and *x* is the distance from the S–L interface.

According to the law of solute conservation, the diffusion flux of Si solute at the front of the S–L interface should be equal, and the following equation can be obtained:

(3) $$C_{L}^{*}=(C_{0}-C_{\mathrm{Si}})\frac{V}{D}x+C_{0}$$

In the Al-Si binary system, due to the very low solid solubility of the Si phase, the solidus is approximately vertical, and the Si phase composition does not change with the composition of the Al-Si alloy. Therefore, the solid phase composition of Si is a straight line, *C*_Si_ = 1. Since the Si phase composition *C*_Si_ is always larger than the initial composition *C*_0_, the Si solute content at the S–L interface front decreases linearly with the growth of the Si phase, so that the liquid phase composition at the S–L interface front is always less than the initial composition of the alloy. For the Si phase with very low solid solubility, the S–L interface front cannot establish a steady-state boundary layer. As the Si phase grows, the Si solute depletion becomes higher, which is completely different from the α-Al phase solid solution. In the Al-40Si alloy, the S–L interface front of the primary Si phase growth produces Al atom enrichment and Si atom depletion.

The eutectic concentration under HP can be expressed as [32]:

(4) $$C_{E}^{P}=C_{E}\exp(\overset{⌢}{e}P)$$

where *C*_E_ is the eutectic concentration of Al-Si alloy at atmospheric pressure, $C_{E}^{P}$ is the eutectic concentration of Al-Si alloy at HP, *P* is the pressure, and $\overset{⌢}{e}$ is a constant.

For Al-Si alloys, *C*_E_ = 12.6 wt.%, $\overset{⌢}{e}$ = 0.195 GPa^−1^. The eutectic point at 3 GPa is about 22.6 wt.%, which is significantly higher than that at atmospheric pressure. The solidification distance *x*_e_ of the primary Si corresponding to the eutectic composition reflects the size of the primary Si. The solidification distance $x_{e}^{P}$ of primary Si under HP can be expressed as:

(5) $$x_{e}^{P}=\frac{D}{V}\frac{C_{0}-C_{E}^{P}}{1-C_{0}}$$

where $x_{e}^{P}$ is the solidification distance of the primary Si under HP.

As can be seen from Equation (5), under HP, the eutectic point of the Al-40Si alloy increases, $C_{0}-C_{E}^{P}$ decreases, and the solute diffusion coefficient *D* decreases exponentially due to the increase in pressure (Figure 4), which causes the solidification distance *x*_e_ of the primary Si to decrease and the primary Si to be refined. From the Al-Si high-pressure phase diagram in Figure 5, it can be seen that the primary Si phase region decreases and the α-Al phase region increases with increasing pressure. Since the solute diffusion coefficient *D* decreases exponentially with increasing pressure, the higher the pressure, the lower the Si solute concentration at the S–L interface front and the higher the Si atom depletion, which inhibits the faceted growth of the Si phase and thus the primary Si boundary shows a non-faceted morphology. Therefore, HP solidification makes the morphology of the primary Si blunt. According to the calculation results in Figure 4, the change in *D* is small when the melt temperature increases from 1273 K to 1373 K at 3 GPa. The size of primary Si changes little according to Equation (5), which is consistent with the experimental results in Figure 3c,d.

At atmospheric pressure, the primary Si in the Al-40Si alloy grows first, and when the Si phase interface front reaches the eutectic temperature, eutectic Al-Si is formed if the liquid phase composition is in the eutectic phase region; if the liquid phase composition is in the α-Al phase region, the α-Al phase will precipitate first depending on the primary Si, and then the eutectic Al-Si will precipitate when the liquid phase composition is in the eutectic phase region. The primary Si is able to grow sufficiently at atmospheric pressure to form large-sized primary Si.

The growth of the primary Si in the Al-40Si alloy at HP is similar to that at atmospheric pressure. Compared to atmospheric pressure, the region of the primary Si in the Al-Si phase diagram is reduced at HP, and the primary Si phase cannot grow sufficiently to refine the primary Si.

### 3.3. Microstructure and Mechanical Properties of SiC/Al-40Si Composite

**Figure 6:**
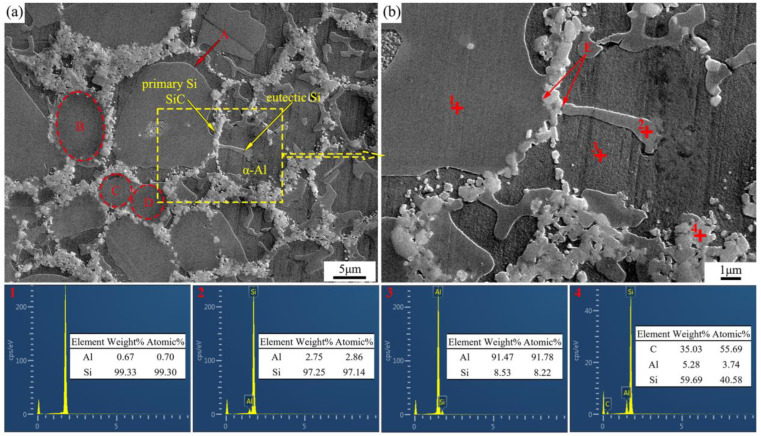
Microstructure of SiC/Al-40Si composite fabricated at 3 GPa. (**a**) Microstructure; (**b**) the selected area in (**a**); (1–4) EDS point analysis in (**b**).

The SiC/Al-40Si composite was chosen to be prepared at 3 GPa because of the best refinement and the smallest volume fraction of the brittle primary Si in the Al-40Si alloy. The microstructure of the SiC/Al-40Si composite prepared at 3 GPa is shown in Figure 6.

The EDS point analysis shows that points 1, 2, 3 and 4 correspond to primary Si, Si, the Al phase and SiC particles, respectively. It can be found that the faceted growth of the large-sized primary Si under HP is inhibited, and the boundary of the primary Si exists in a certain curvature, as shown by the arrow at A in Figure 6a. Since the SiC particles (0.1–1μm) are much smaller than the Al-40Si alloy powder (9–11 µm), the SiC particles are distributed in the voids around the alloy powder after mixing and cold pressing. The high viscosity of the alloy melt under HP conditions makes it difficult for the SiC particles to move and maintain the initial network distribution basically. Both primary Si and eutectic Si can be grown in attachment to SiC particles, as shown by the arrow at E in Figure 6b. According to the analysis in Figure 5, the presence of SiC particles at the front of the growing S–L interface of the primary Si will hinder the continued growth of the primary Si, and eventually the SiC-Si spherical microstructure is formed by the primary Si and the network distributed SiC, as shown by the dashed lines B, C and D in Figure 6a. The primary Si in the Al-40Si alloy is a long strip (Figure 3c), while the primary Si is separated into spherical shapes by the addition of SiC (Figure 6a), which avoids the adverse effect of large sizes of primary Si on the mechanical properties.

**Figure 7:**
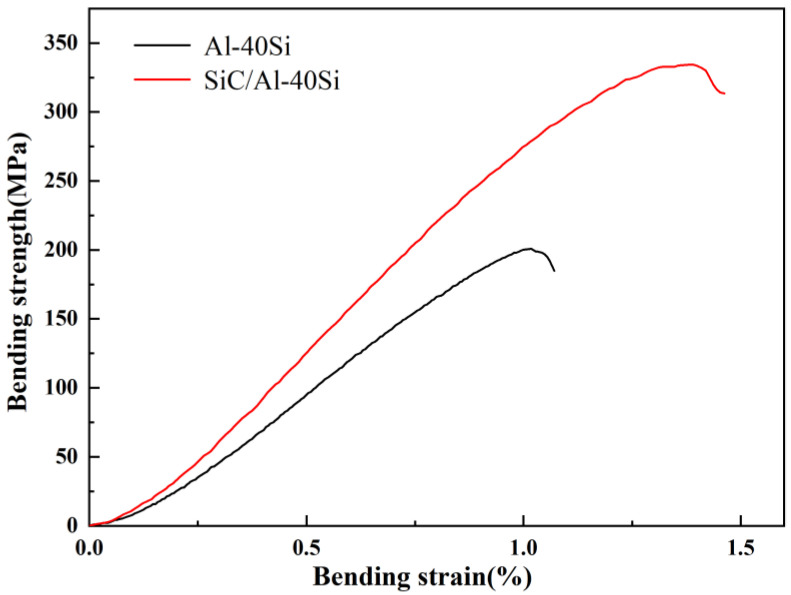
Results of the bending tests of materials fabricated at 3 GPa.

Figure 7 shows the results of the bending tests of materials. The bending strength of SiC/Al-40Si composite prepared via high-pressure solidification is 334 MPa, which is 66% higher than that of the matrix alloy. Furthermore, the bending strain of the SiC/Al-40Si composite is 1.39%, which is 36% higher than that of the matrix alloy.

**Figure 8:**
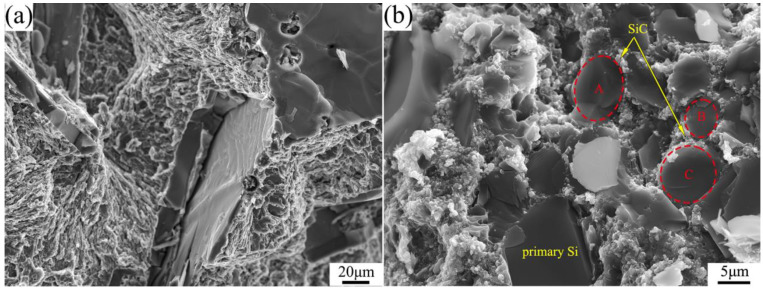
Bending fractographies of materials. (**a**) Al-40Si; (**b**) SiC/Al-40Si.

Figure 8 shows the bending fractographies of the materials fabricated at 3 GPa. It can be seen that the lower bending strength of the Al-40Si alloy is due to the brittle cleavage fracture of the primary Si phase. The SiC/Al-40Si composite has higher bending strength and strain, which is related to the SiC-Si spherical microstructure formed by primary Si and SiC reinforcement. The SiC-Si spherical microstructure makes the primary Si change from overall brittle cleavage fracture to brittle fracture of multiple regions, as shown in the dotted lines A, B and C in Figure 8b. This indicates that the interface bonding strength between SiC particles and primary Si is high enough to change the direction of crack propagation.

Compared to the Al-40Si alloy, the strengthening effect of the added SiC particles is divided into two aspects—one is the strengthening of the Al matrix and the other is the strengthening of the primary Si.

The strengthening of the Al matrix by SiC particles is due to the large difference in thermal expansion between the SiC particles and the α-Al matrix, resulting in a large number of dislocations in the matrix near the Al/SiC interface and resulting in strengthening. It can be calculated according to the following equation [33]:

(6) $$\Delta \sigma_{\mathrm{CTE}}=\beta G_{m}b\sqrt{\rho^{\mathrm{CTE}}}$$

(7) $$\rho^{\mathrm{CTE}}=\frac{A_{\rho }\Delta \alpha \Delta TV_{p}}{2br(1-V_{p})}$$

where *β* is a constant of about 1.25, *G*_m_ is the shear modulus of the matrix, *b* is the Berger vector, *ρ*^CTE^ is the dislocation density caused by the mismatch of the coefficient of thermal expansion, *A_ρ_* is a geometric constant that varies between 10 and 12 depending on the geometry of the particles, Δ*α* is the difference in the coefficient of thermal expansion, Δ*T* is the difference between the preparation temperature and the test temperature, and *r* is the average radius of the particles.

For Al matrix, *G*_m_ = 28 GPa, *b* = 0.286 nm [34]. for SiC particles, *r* = 0.5 μm, *A_ρ_* = 12; *α*_SiC_ = 3.5 × 10^−6^/K, *α*_Al_ = 22.6 × 10^−6^/K [10,35]. The preparation temperature is 1273 K and the test temperature is 298 K. The volume fraction of ex situ SiC is 20 vol.%, but part of SiC is in contact with Si phase. According to the volume ratio of Al and Si in the Al-40Si alloy, assuming the volume fraction of SiC in contact with Al phase is 11 vol.%, we can obtain Δ*σ*_CTE_ = 98.4 MPa. The dislocation strengthening between Si/SiC is neglected because the difference in thermal expansion between SiC particles and Si phase is small.

The strengthening of primary Si by SiC particles is due to its hindering effect on cracking. The fracture stress can be expressed as [36]:

(8) $$\sigma_{f}=\frac{1}{Y}\sqrt{\frac{2E\gamma }{\pi C}}$$

where *C* is the crack size, *γ* is the fracture surface energy, *E* is the Young’s modulus and *Y* is the dimensionless correction factor.

**Figure 9:**
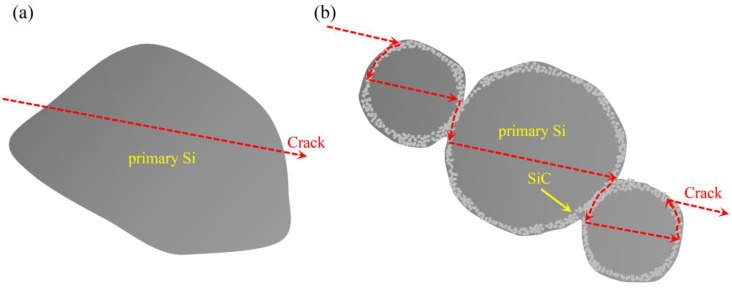
Schematic illustration of crack propagation. (**a**) Primary Si phase; (**b**) SiC-Si spherical microstructure.

Based on the fracture morphology of Figure 8, a schematic diagram of the crack propagation of the primary Si and SiC-Si spherical microstructures is drawn, as shown in Figure 9. As can be seen from Equation (8), primary Si fractures along the cleavage face, with lower crack extension resistance and fracture energy, resulting in lower alloy properties. When the SiC-Si spherical microstructure breaks, SiC particles change the direction of crack propagation, increase the crack propagation resistance and fracture energy, and make the fracture path of primary Si longer, so as to improve the strength of the composite.

## 4. Conclusions

In this study, the microstructure evolution mechanism of primary Si in Al-40Si alloys solidified under HP was studied. Then, the SiCp/Al-40Si composite was prepared via the addition of SiC particles under high-pressure solidification conditions, and its mechanical property and strengthening mechanism were studied. The conclusions are as follows:

(1) As the pressure increases from 1 atm to 3 GPa, the primary Si phase in Al-40Si alloy is refined. With increasing pressure, the composition of the eutectic point increases, the solute diffusion coefficient decreases exponentially, and the concentration of Si solute at the front of the S–L interface of the primary Si is low, which contributes to the refining of the primary Si phase and inhibiting its faceted growth.

(2) In the SiC/Al-40Si composite prepared at 3 GPa, the primary Si phase and the SiC reinforcement form a SiC-Si spherical structure, which makes the primary Si change from the overall brittle cleavage fracture to the multi-region brittle fracture, increasing the crack propagation resistance and path, and improving the strength of the composite.

(3) The bending strength of SiC/Al-40Si composite prepared at 3 GPa was 334 MPa, which was 66% higher compared to the Al-40Si alloy prepared under the same pressure.

## Data Availability

All data and models used during the study appear in the submitted article.
